# The Genus* Luehea* (Malvaceae-Tiliaceae): Review about Chemical and Pharmacological Aspects

**DOI:** 10.1155/2016/1368971

**Published:** 2016-10-13

**Authors:** João Tavares Calixto-Júnior, Selene Maia de Morais, Aracélio Viana Colares, Henrique Douglas Melo Coutinho

**Affiliations:** ^1^Juazeiro do Norte College (FJN), Juazeiro do Norte, CE, Brazil; ^2^Biotechnology Postgraduation Programme (RENORBIO), Laboratory of Natural Products, State University of Ceará, Itaperi Campus, Fortaleza, CE, Brazil; ^3^UNILEAO University Center, Juazeiro do Norte, CE, Brazil; ^4^Department of Biological Chemistry, Regional University of Cariri, Crato, CE, Brazil

## Abstract

Popularly known as “açoita-cavalo” (whips-horse),* Luehea* species (Malvaceae-Tilioideae) are native to America and are used in folk medicine as anti-inflammatory, antidiarrheal, antiseptic, expectorant, and depurative and against skin infections. Although there are studies showing the chemical constituents of some species, the active substances have not been properly identified. A systematic study was carried out through a computer search of data on CAPES journals, SciELO, ISI Bireme, PubMed, ScienceDirect, ScienceDomain Medline, and Google Scholar from published articles using key words:* Luehea*, açoita-cavalo, and Malvaceae.* Luehea divaricata* was the species with the highest number of studies observed. Triterpenes (9), flavonoids (6), and steroids (4), including saponins, organic acids (4), and one lignan, are the main types of secondary metabolites registered and the most cited flavonoids were rutin and quercetin and among triterpenes there was maslinic acid, which might be associated with the popular indication of its anti-inflammatory action. The vitexin, a C-glycosylated flavone, isolated from three different species, is cited as a possible taxonomic marker of the genus. Studies confirm in part the medicinal uses of plants named as “açoita-cavalo” species. Some pharmacological activities, not assigned to the species of the genus* Luehea* by populations, were observed in laboratory experiments.

## 1. Introduction

Malvaceae is a family consisting of herbs, subshrubs, shrubs, lianas, and small and large trees, with about 250 genera and 4,200 species, of which, in Brazil, about 80 genera and 400 species are found [1]. According to the List of Species of Flora of Brazil, 69 genera and 754 species are indicated, with 30 genera distributed in 393 taxa of the subfamily Malvoideae [2, 3].

*Luehea* genus, which belongs to Malvaceae family, is essentially neotropical, existing in southern Mexico, including the West Indies to Uruguay and Argentina. Currently there are about 25 species and 3 varieties, of which 12 species and one variety exist in Brazil, with its highest concentration in the southeast and midwest regions [4]. The genus* Luehea* is present in Brazilian Cerrado and thirteen species are registered in Brazilian herbariums:* Luehea altheaeflora* Spruce ex Benth.,* Luehea fiebrigii* Burret,* Luehea candicans* Mart.,* Luehea candida* (Moc. & Sessé ex DC.) Mart.,* Luehea crispa* Krapov.,* Luehea cymulosa* Spruce ex Benth.,* Luehea divaricata* Martius et Zuccarini,* Luehea grandiflora* Mart. & Zucc.,* Luehea paniculata* Mart. & Zucc.,* Luehea ochrophylla* Mart.,* Luehea rosea* Ducke and* Luehea speciosa* Willd., and* L. conwentzii* K. Schum.

The lectotype of* L. conwentzii* was selected and a new synonym was proposed—*L. eichler* Schum [5]. To Rizzini and Mors [6], the various species are very similar to each other, getting the same common names (horse-whips) and having identical uses.

Among the species of this genus, the most cited are* L. grandiflora* and* L. divaricate*, popularly known as “açoita-cavalo” (whips-horse) [7]. The closest species of* L. divaricata* is* L. paniculata*, which is a tree a little lower, existing in Bolivia, Paraguay, Peru, and several states of Brazil [8]. The herbarium specimens of* L. grandiflora* showed close similarities to those of* L. speciosa* because they are both very polymorphic. However,* L. speciosa* staminodia are profoundly fimbriated, while those of* L. grandiflora* are slightly fimbriated in the apex. So* L. grandiflora* was considered a synonym of* L. speciosa* but was later rehabilitated [9].

A* Luehea* herbal product, composed of dried leaves, is used against dysentery, leucorrhoea, rheumatism, gonorrhea, and tumors; infusion of the flowers is used against bronchitis and the root is depurative [10]. Aerial parts of* L. divaricata* are used popularly for skin wounds and for intimate hygiene [11]. Backes and Irgang [12] reported the use of the bark of this plant as antirheumatic, antidiarrheal, antiseptic, expectorant, and cleanser.

The current state of knowledge of the chemistry and pharmacology of genus* Luehea* indicates its potential for developing anti-inflammatory drugs and antibiotics; however, few studies with species of this genus were reported.

Due to the importance of the genus* Luehea*, as a source of new medicinal agents, a review is worthwhile. The data may support future multidisciplinary studies involving phytochemical studies of the genus species to perform a critical analysis of their use by populations with a view to the preservation of plant species in their respective biomes and promoting rational use of “açoita-cavalo” as a therapeutic resource. A systematic study was carried out through a computer search of data on CAPES journals, SciELO, ISI Bireme, PubMed, ScienceDirect, ScienceDomain Medline, and Google Scholar from published articles using key words:* Luehea*, açoita-cavalo, and Malvaceae. Data was also obtained from theses, proceedings, and book of abstracts and reviews indexed in the databases, used for this work, as well as abstracts and full papers published in scientific events. Dissertations and doctoral theses of Brazilian and international students with relevant data were also examined. Data presented include scientific name and activities of the plants.

## 2. Development

### 2.1. Chemical Aspects

#### 2.1.1. *Luehea divaricata*

Little information is known about the chemical constituents present in the genus* Luehea* [8]. The phytochemical analysis of the leaves of this species showed mainly the presence of flavonoids, saponins, and catechin tannins (condensed tannins) [13]. To a lesser extent, the authors cited the presence of alkaloids, fixed oils, anthocyanins, carotenoids, and polysaccharides. The species presents tannins, essential oil, resin, and mucilage [14].

Portal et al. [15], conducting a phytochemical screening on the extract of* L. divaricata* collected in Belém, Pará State, Northern Brazil, noted the presence in the leaves of reducing sugars, proteins, amino acids, tannins, catechins, flavonoids, carotenoids, steroids, triterpenoids, and saponins. Several authors performed a phytochemical screening with ethanol extracts of leaf and stem and also observed the presence of flavonoids, tannins, saponins, and triterpenes/steroids [16–18] and Bertucci et al. [19] screening native species of the Uruguay River observed in the hydroalcoholic extract of leaves of* L. divaricata* the presence of these same constituents.

**Figure 1:**
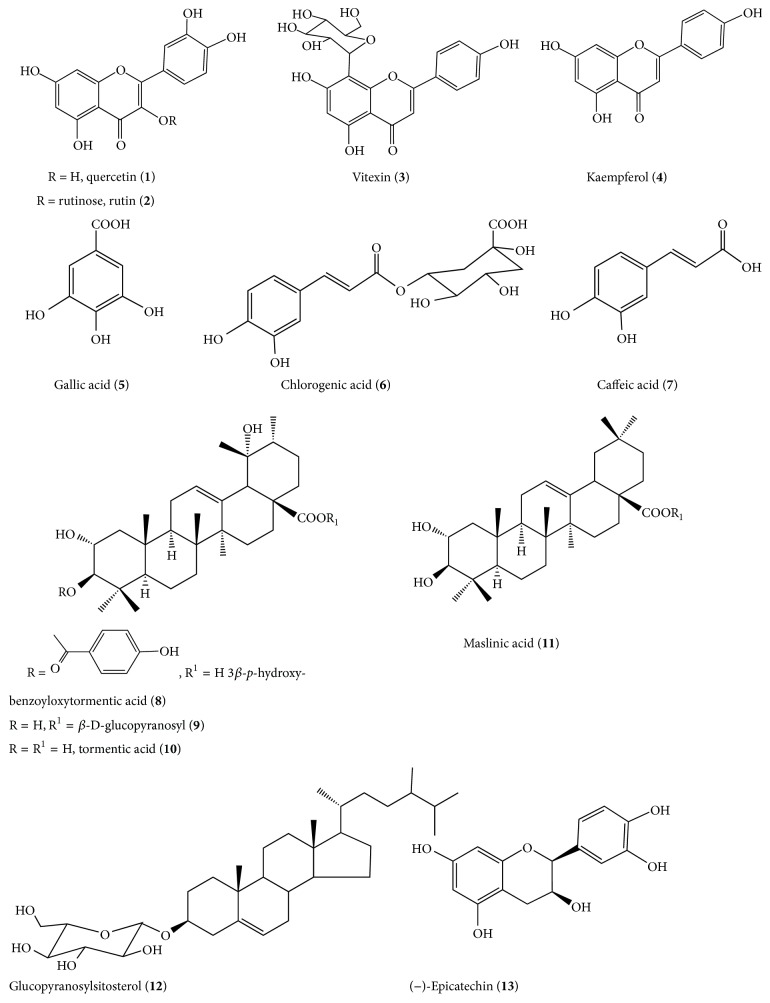
Phytocompounds isolated from* Luehea divaricata*.

Lopes [18], through phytochemical study of* L. divaricata* collected in Southern Brazil, identified from the alcoholic extract of leaves, tannins, saponins, and flavonoids and isolated by two-dimensional thin-layer chromatography the flavonoids quercetin (**1**), rutin (**2**), and vitexin (**3**) (Figure 1).

Vargas et al. [20] also point out the presence of tannins, flavonoids, and saponins in leaves and bark of* L. divaricata* and also the presence of quercetin (**1**) and kaempferol (**4**) in the extracts. However, Maraschin-Silva and Aqüila [21], investigating the allelopathic potential of this species, only infer about the presence of tannins and saponins in aqueous leaf extract.

Arantes [17] analyzed the ethanol extract of leaves collected in Southern Brazil by HPLC and reported the presence of the flavonoids quercetin (**1**), rutin (**2**), and kaempferol (**4**) and aromatic acids gallic acid (**5**), chlorogenic acid (**6**), and caffeic acid (**7**) (Figure 1).

Tanaka et al. [22] isolated from the methanol extract of leaves of* L. divaricata*, collected in Maringá, Paraná State, Southern Brazil, a new triterpene (basic skeleton of ursene): 3*β*-*p*-hydroxybenzoyloxytormentic acid [3*β*-(*p*-hydroxybenzoyloxy)-2*α*-hydroxyurs-12-en-28-oic acid] (**8**), a mixture of tormentic acid ester glucoside (**9**), tormentic acid (**10**), and the maslinic acid (olean-12-ene-2*α*,3*β*-diol) (**11**) (basic skeleton of oleanane) (Figure 1).

Tanaka et al. [10], in continuation of previous work, isolated from stem bark and leaves, besides the mentioned triterpenes, the steroid glucopyranosylsitosterol (**12**), (−)-epicatechin (**13**), a flavonoid which belongs to a class of flavan-3-ol, and vitexin (**3**), as already indicated in the same species isolated in previous work [18] (Figure 1).

The presence of flavonoids such as vitexin (**3**) [23, 24] and triterpenes as maslinic acid (**11**) [25, 26] may be associated with the popular indication of its anti-inflammatory action [27].

Besides polyphenols (flavonoids, catechins, anthocyanins, and tannins) the phytochemical screening of the hydroalcoholic extract of bark of* L. divaricata* collected in Leme, São Paulo State, Southeastern Brazil, identified the presence of saponins, triterpenes, steroids, and anthracenes [28]. Walker et al. [29], in histochemical and morphoanatomical study of leaves of* L. divaricata* Mart., reported about the presence of mucilage and calcium oxalate in the form of a prism and drusen in idioblasts.

**Figure 2:**
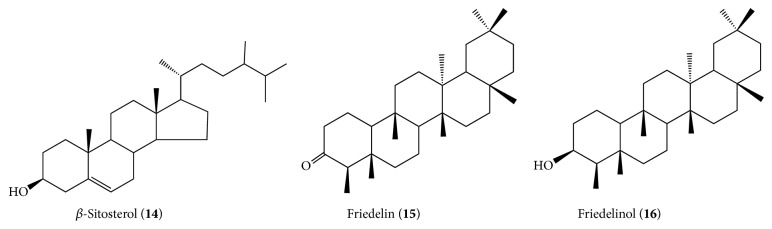
Phytocompounds isolated from* Luehea ochrophylla*.

*Luehea ochrophylla.* Two papers that focus on the phytochemical analysis of this species are listed in this review. The phytochemical study of skins of* L. ochrophylla* collected in Grão Mogol, Minas Gerais State, Southeastern Brazil, resulted in the isolation of hydrocarbons, aliphatic esters of steroid *β*-sitosterol (**14**), and pentacyclic triterpenes friedelin (**15**) and *β*-friedelinol (**16**) [30] (Figure 2).

#### 2.1.2. *Luehea grandiflora*

**Figure 3:**
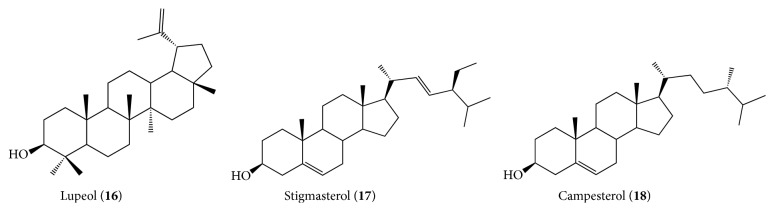
Phytocompounds isolated from* Luehea grandiflora*.

The works of da Silva et al. [30] that indicate polyphenols in the cortex of* L. grandiflora* and Rosa et al. [31, 32] were the only works with focus on the phytochemistry of this species. Rosa et al. [31, 32] working with crude ethanol extract of leaves collected in Goiás State, Brazil, observed, from fractionation by column chromatography isolation, in chloroform fraction, the lupeol triterpene (**16**) and the mixture of steroids *β*-sitosterol (**14**), stigmasterol (**17**), and campesterol (**18**) (Figure 3).

#### 2.1.3. *Luehea candida*

**Figure 4:**
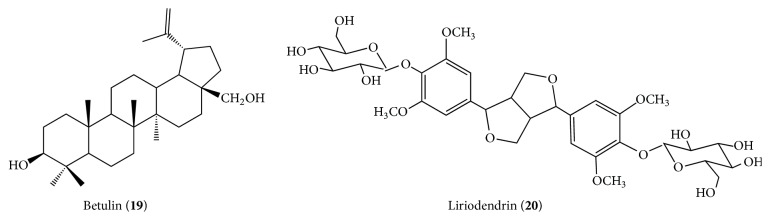
Phytocompounds isolated from* Luehea candida*.

The anticancer potential of* L. candida* extracts was evaluated against many cell lines and lupeol (**16**), betulin (**19**), (−)-epicatechin (**13**), vitexin (**3**), and a lignan liriodendrin (**20**) were isolated from the active fractions [8] (Figure 4).

Saénz and Nassar [33] established the presence, in small quantities, of alkaloids in this species. Alkaloids were also pointed out in qualitative studies on* Luehea seemannii* collected in Eastern Nicaragua [34] and leaves and bark in this species collected in Villa Neily, Costa Rica [35, 36].

#### 2.1.4. *Luehea paniculata*

Barbosa and Reed [35, 36] analyzing ethanol extracts of* L. grandiflora* and* L. paniculata* collected in Goiania, Brazil, inferred about the presence of tannins and flavonoids in leaves and bark of both species. The existence of rutin (**2**) in* L. paniculata* was identified by means of thin-layer chromatography.

**Figure 5:**
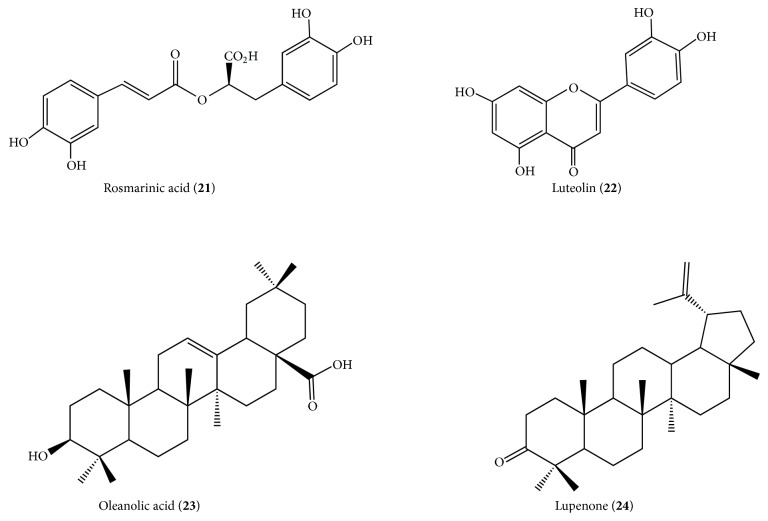
Phytocompounds isolated from* Luehea paniculata*.

Calixto Júnior et al. [37] observed by HPLC-DAD fingerprinting in* L. paniculata* ethanolic extracts (leaves and sapwood) the presence of the gallic acid (**5**), chlorogenic acid (**6**), and rosmarinic acid (**21**) and flavonoids rutin (**2**), vitexin (**3**), and luteolin (**22**) (Figure 5).

Alves et al. [38] reported the isolation of triterpenes maslinic acid (**11**) from the chloroform fraction and oleanolic acid (**23**) and lupenone (**24**) from the ethyl acetate fraction, as well as evaluating the antiproliferative activity of the crude extract fractions of ethyl acetate and hydromethanol obtained from the leaves of* L. paniculata* collected in the Cerrado, Central Brazil (Figure 5).

#### 2.1.5. *Luehea candicans*

Silva [39], in a study that involved extraction, isolation, and identification of the chemical constituents of the branches and leaves of* L. candicans* in the Puerto Rico region, Paraná State, Southern Brazil, got the isolation, the crude methanol extract of the stems of two triterpenes belonging to the class of lupins, the lup-20(29)-en-3*β*-ol (lupeol) (**16**) and the betulin—3*β*,28-diidroxilup-20(29)-ene (**19**). The works of Silva [39] and Da Silva et al. [8] were the only ones found on chemical research in this species.

The presence of vitexin in the leaves of* L. candicans*,* L. divaricata*, and* L. paniculata* species can suggest the hypothesis that this flavone may be a possible taxonomic marker of the genus* Luehea*.

### 2.2. Ethnopharmacological and Pharmacological Aspects

The popular knowledge about the medicinal uses of horse-whips is reported by several authors [40]; however, there are few studies available on the pharmacological potential of the species [12]. The highest number of citations on ethnopharmacological information is assigned to the species* L. divaricata*; however, some other species appear in other surveys.* Luehea seemannii* is popularly used against bites and stings (snake, scorpion, and insects) and also as an astringent in Eastern Nicaragua [34].

In a survey conducted about ethnopharmacology of medicinal plants of the Pantanal (Mato Grosso State, Brazil),* L. divaricata* is among the species with the highest relative importance value (1.50) between values ranging from 0.17 to 1.87, among the 261 species mentioned in the work [27]. The authors report citations, by respondents, about the use of* L. divaricata* in the treatment of lung and upper respiratory disease. However, they stress that no scientific evidence exist on the activity of the species in the regulation of cough, while its antibiotic properties also vary. Bessa et al. [41] reported a quote from “açoita-cavalo” as being of use to the treatment of ulcer in rural community of Green Vale Settlement, Tocantins State, Central Brazil.

Several other reports of uses of this species were found: treatment of rheumatism; arthritis; dysentery; internal cleaning of wounds and ulcers; depurative; sore throat; painkiller for toothache; astringent; evils of the bladder; sleep balance; melena (intestinal colic followed by diarrhea with painful bowel movements and presence of blood in the stool); leucorrhea; gonorrhea; bleeding; cough; laryngitis; bronchitis; deworming; gastritis; poor digestion; diarrhea; and cancer and tumors [9, 42–44].

Degen et al. [45] reported medicinal plants traded by their common names and cited “Francisco Álvarez” as* L. divaricata* being employed against diabetes in Argentina and in Paraguay, where the same common name matches* the Banara arguta* Brig. (Flacourtiaceae). The term “Francisco Álvarez” as reference to “açoita-cavalos” in Paraguay is also cited by Carvalho [46], which emphasizes the use of other common names abroad: azota caballo and árbol de San Francisco, Argentina; Francisco Álvarez, Uruguay; and ka'a oveti, Paraguay. In various regions of Brazil the author highlights açoita: açoita-cavalo-do-miúdo, açoita-cavalo-branco, ivantingui, and vatinga in the São Paulo State; açoita-cavalo in Paraná State; in the states of Rio de Janeiro and São Paulo, biatingui, erviteira-do-campo, estibeiro, and estriveira; in Bahia State and São Paulo State, guaxima-do-campo, ibatingui, and ivatingui; in Minas Gerais State, ivitinga; in Bahia State, ivitingui, luitingui, mutamba, and pau-de-canga; in Santa Catarina State, salta-cavalo; in Paraná State and São Paulo State, soita and soita-cavalo; and, in Paraná State, ubatinga.

In folk medicine the “açoita-cavalo” is used in cases of dysentery, bleeding, arthritis, leucorrhoea, rheumatism, and tumors [41]. In ethnodirected work at Quilombo Sangrador, Maranhão State, Northeastern Brazil, the “açoita-cavalo” was one of the species with more valuable importance, with indications for the treatment of diseases of the genitourinary system [47]. Basualdo and Soria [48] included* L. divaricata* Mart. in the list of medicinal plants of Paraguay used in fighting respiratory infections.

The leaves of* Luehea* are marketed as herbal against leucorrhoea, rheumatism, gonorrhea, and tumors; infusion of the flowers is used against bronchitis and the root is depurative [10]. Rai [11] reports that the aerial parts of “açoita-cavalo” (*L. divaricata* Martius et Zuccarini) are used in traditional medicines for skin wounds, grain cleaning, and vaginal washings. Maffei [49] in work on medicinal plants of Uruguay and Chiriani [50] in study about phytotherapy in Argentina inform that the barks are used as antipyretic and antianemic agents, in addition to antidiarrheal, astringent, and antitumor. Quotes on the leaves as herbal against rheumatism, dysentery, gonorrhea, soothing, and antispasmodic are observed in several works [10, 49, 51–53]. The root is pointed out as anti-inflammatory and cleansing by Alice et al. [54], and the flowers are cited, along with bark and leaves, with diuretic, antiarthritic, antileucorrhoea, and wound healing of the skin and vaginal washings [22, 40, 49, 55, 56]. The bath of the stem bark was quoted in ethnobotanical survey in the Upper Rio Grande, Minas Gerais State, Southeastern Brazil, [57] as being of use for dysentery, as antirheumatic and antihemorrhagic agent.

According to Alice et al. [58], the bark of the* L. divaricata* is also used to combat the fever and is also suitable for gastrointestinal and liver disorders. The leaves are used as anti-inflammatory and employed in disorders of the respiratory tract and bronchitis. In popular phytotherapy the cortex of stems of* L. divaricata* is used in decoctions and infusions by tonic and antidiarrheal characteristics [59]. The resin of the fruit is applied as antiodontalgic; 1% flowers infusion is used as sedative and the infusion of the leaves as anti-inflammatory [60, 61].

### 2.3. Microbiological Activities

Five studies have reported effects against microorganisms in this species. The antifungal activity of* L. divaricata* was evaluated by Zacchino et al. [62]. As a result, the authors pointed out the moderate action of dichloromethane extract in inhibiting the growth of hyphae of some species of dermatophytes; however, this inhibitory effect is not observed for other fungal species [10, 40]. However, the extract of* L. divaricata* showed strong inhibition of the growth of* Staphylococcus aureus*,* Staphylococcus epidermidis*,* Klebsiella pneumonia*, and* Escherichia coli* in one study [40] but showed only moderate inhibition in another study and another collection site [10].

Montovani et al. [63] also investigated antimicrobial action in extract of* L. divaricata*. Collected in São Pedro do Iguaçu, Paraná State, Southern Brazil, the authors obtained extracts of leaves from three solvents (hexane, ethyl acetate, and ethanol) and diagnosed positive effects in inhibiting* S. aureus*,* Bacillus cereus*,* E. coli*, and* Salmonella typhi*. However, for the fungus* Aspergillus niger*, they obtained negative result; Coelho De Souza et al. [64], as a result of ethnopharmacological study, evaluated the antimicrobial potential of some plants in common use in the Rio Grande do Sul State (Southern Brazil) using the agar diffusion method; the authors found that the methanol extract of* L. divaricata* showed activity against the* Micrococcus luteus* bacteria. The extract showed no activity against other bacteria such as* S. aureus*,* S. epidermidis*, and* E. coli*, even against yeast* Candida albicans*.

Marques et al. [65] reported the biological activities study of* L. paniculata* of the Brazilian Cerrado, which showed inhibitory effect of ethanol extract of* L. paniculata* leaves on* S. aureus* bacteria.

Calixto Júnior et al. [37] pointed out as irrelevant the activity of* L. paniculata* leaf ethanolic extract (LEELP) tested against six strains of* Candida* (MIC ≥ 1024). This work is the first recorded piece of research on the potentialization of Fluconazole using extracts of* L. paniculata* against three strains of* Candida*, in particular,* C. tropicalis* and* C. albicans*, and there was, therefore, a synergism when the extracts were combined with Fluconazole.

### 2.4. Anti-Inflammation and Toxicity

Lopes [18] investigated the anti-inflammatory action of* L. divaricata*. From an experiment using the aqueous extract obtained from the dried leaves of the plant, the authors used the test of rat paw edema induced by carrageenan. The extract was administered intraperitoneally at doses of 100 and 150 mg/kg and orally, using a stomach tube at a dose of 300 mg/kg. According to the authors, the dose of 150 mg/kg administered intraperitoneally obtained the peak reduction of the edema. Siqueira [28] claims to have the hydroalcoholic extract of* L. divaricata* that reduced the rate of ulcerative lesions produced by indomethacin and ethanol. The author indicates that the mechanism of action antiulcerogenic is partly related to the activity of sulfhydryl radicals and by the precipitation of proteins produced by the presence of polyphenols present in the plant.

Bianchi [66] conducted studies of acute and subacute toxicity with extracts of this species. In both tests Swiss mice, males, were used. The alcoholic extract administered intraperitoneally at doses of 250 mg/kg and 500 mg/kg triggered diarrhea and bristling fur, within 72 hours, that were observed in the acute toxicity test. 50% of deaths of the animals were seen using a dose of 500 mg/kg for 48 hours. In the subacute toxicity test, the authors administered a dose of 25 mg/kg of aqueous and alcoholic extract by means intraperitoneally, once a week for 8 days. The aqueous extract triggered the death of an animal after the 8th dose, unlike the alcoholic extract that at this dose did not cause any deaths [67]. Vargas et al. [20] showed genotoxic (mutagenic) activity in aqueous leaf extract of* L. divaricata* in the Ames test (*Salmonella*/microsome) with microsomal activation. This fact can be explained by the presence of tannins and flavones in the leaves of the species that present patterns of hydroxylation, which could possibly cause damage to the DNA structure [54].

The phytotoxic activity of* L. divaricata* was shown clearly by Souza et al. [68]. The authors point out the test with aqueous extract of leaves collected in Pelotas, Southern Brazil, which showed an inhibitory effect on the germination of* Lactuca sativa*. Nevertheless,* L. divaricata* extracts demonstrate* in vivo* lack of toxicity or mutagenicity [69, 70].

### 2.5. Antioxidant Potential

Good antioxidant activity and analgesic properties in leaves of* L. divaricata* were pointed out by Müller [40]. By DPPH method, the author observed the crude extract and ethyl acetate and butanol fractions with similar antioxidant with the quercetin. Arantes [17] showed antioxidant activity at low concentrations of ethanol extract of the leaves of this species, observed by reducing basal levels of lipid peroxidation by the extract and its protective effect against lipid peroxidation and decreased cell viability induced by nitroprusside sodium in rat brain* in vitro*.

Antioxidant effect of the ethanol extract of* Luehea paniculata* roots was evaluated by the method of scavenging free radical DPPH [37]. This extract demonstrated an antioxidant potential almost similar to the value associated with the flavonoid quercetin (EC_50_ = 0.85 and 0.01 mg/mL, resp.). Calixto Júnior et al. [37] showed good results in antioxidant test of leaf extract and bark with IC_50_ values: 0.32 and 0.24 mg/mL, respectively.

### 2.6. Antiproliferative Activity

Alves et al. [38] analyzed the antiproliferative activity of the crude extract and ethyl acetate and hydromethanol fractions of* L. paniculata* leaves. The evaluation was performed at concentrations from 0.25 to 250 mg/mL against three human tumor cell lines, breast (MCF-7), lung (NCI-H460), and glioma (U251), by the colorimetric method with sulforhodamine B. The results showed moderate antiproliferative potential for those fractions.

The crude methanolic extracts of the branches and leaves of* Luehea candicans* were evaluated using the following cancer cell lines: MCF-7 (breast), NCI-ADR (breast expressing the multidrug resistance phenotype), NCI-460 (lung), UACC-62 (melanoma), 786-0 (kidney), OVCAR (ovarian), PCO-3 (prostate), HT-29 (colon), and K-562 (leukaemia). The crude methanolic extracts from the branches (B) and leaves (L) were able to inhibit the growth of the K-562 and 786-0 cell lines in a dose-dependent manner, with GI_50_ values of 8.1 and 5.4 *µ*g /mL, respectively. The hexane (L1), chloroform (L2), and methanol (L4) fractions derived from extract L showed a high selectivity and pronounced cytostatic activity against 786-0 (GI_50_~40 *µ*g /mL). A significant amount of lupeol was isolated from fraction L2. The chloroform (B2) and methanol (B3) fractions derived from extract (B) exhibited less selectivity, showing the highest cytostatic activity against K-562, NCI-ADR, OVCAR, MCF-7, and NCI-460 cells, with GI_50_ values between 27 and 40 *µ*g/mL [8].

Silva [39] studied* L. candicans* in the region of Puerto Rico, Paraná State, Southern Brazil, and assessed, by biological tests, the antibacterial, antifungal, and antiproliferative crude extracts, fractions, and isolated compounds. None of the extracts, however, showed significant antibacterial activity; however, the crude extract of the leaves showed antifungal activity against* Candida krusei* strain with a fungicide at a concentration of 125 mg/mL (CMF) and fungistatic concentration of 62.5 mg/mL (CIM). The crude extract of* L. candicans* still showed antiproliferative activity with cytostatic effect of 85% (25 *μ*g/mL) and cytocidal effect by 25%, the highest concentration used for cell kidney cancer. The hexane, CHCl_3_, and MeOH fractions demonstrated a cytostatic effect of 100% against kidney cancer cells. The crude extract of the stems showed cytostatic and antiproliferative activity with little selectivity among the studied strains (25% cell death at the highest concentration) effects, since the crude extract of* L. candicans* and hexane, CHCl_3_ and MeOH fractions were selective for cells of kidney cancer exhibiting antiproliferative activity with cytostatic effect (growth inhibition) by 85% at a concentration of 25 mg/mL for the crude extract and 100% (250 *μ*g/mL) for fractions. The antifungal activity of triterpenes, steroids, and flavonoids isolated was also tested, but without any substances presenting activity.

## 3. Conclusion

Studies confirm in part the medicinal uses of plants named as “açoita-cavalo” species. Some pharmacological activities not assigned to the species of the genus* Luehea* by populations were observed in laboratory experiments.

28 papers focusing on phytochemical studies with species of the genus were observed. The most studied in this regard are* L. divaricata*,* L. paniculata*, and* L. candicans*. The chemical composition of the leaf and stem is similar in different species, leading to the assertion that similar actions relate to common components in different representatives of the genus. Several secondary metabolites were found as triterpenes, steroids, flavonoids, lignans, organic acids, and flavone vitexin.

The vitexin, a C-glycosylated flavone, isolated from three different species of* Luehea* is cited as a possible marker of taxonomic genus and to prove this hypothesis further chemical research studies are required to find it in other species. The flavone vitexin and triterpene maslinic acid may be associated with anti-inflammatory action, which is considered the main popular indication for the genus.

## References

[1] Souza V. C., Lorenzi H. (2005). *Botânica Sistemática: Guia Ilustrado para Identificação das Famílias de Angiospermas da Flora Brasileira*.

[2] Bovini M. G. (2010). Malvaceae na reserva rio das pedras, mangaratiba, Rio de Janeiro, Brasil. *Rodriguesia*.

[3] Bovini M. G., Esteves G., Duarte M. C. (2013). Malvaceae. *Lista de Espécies da Flora do Brasil*.

[4] Cunha M. C. D. S. (1985). Revisão das espécies de gênero *Luehea* Willd. (Tiliaceae) ocorrentes no Estado do Rio de Janeiro. *Sellowia*.

[5] Cunha M. C. D. S. (1982). Contribution to the study of the genus *Luehea* in Brazil a new synonym for *Luehea conwentzii*. *Bradea*.

[6] Rizzini C. T., Mors W. B. (1976). *Botânica Econômica Brasileira*.

[7] Pio Corrêa M. P. (1984). *Dicionário das Plantas Úteis do Brasil e das Exóticas Cultivadas*.

[8] Da Silva D. A., Alves V. G., Franco D. M. M. (2012). Antiproliferative activity of *Luehea candicans* Mart. et Zucc. (Tiliaceae). *Natural Product Research*.

[9] Brandão M. (1991). Plantas medicamentosas do cerrado mineiro. *Informe Agropecuário*.

[10] Tanaka J. C. A., da Silva C. C., Dias Filho B. P., Nakamura C. V., de Carvalho J. E., Foglio M. A. (2005). Chemical constituents of *Luehea divaricata* Mart. (Tiliaceae). *Química Nova*.

[11] Rai M. K. (2003). *Plant-Derived Antimycotics*.

[12] Backes P., Irgang B. (2002). *Árvores do Sul: Guia de Identificação e Interesse Ecológico*.

[13] Bortoluzzi R. C., Walker C. I. B., Manfron M. P. Análise química qualitativa e morfo-histológica de Luehea divaricata Mart.

[14] Lorenzi H. (1988). *Árvores Brasileiras: Manual de Identificação e Cultivo de Plantas Arbóreas Nativas do Brasil*.

[15] Portal R. K. V. P., Lameira A. O., Ribeiro F. N. S. (2013). Fenologia e Screening fitoquímico do Açoita-cavalo. *Manaus, 17° Seminário de Iniciação Científica e 1° Seminário de Pós-Graduação da Embrapa Amazônia Oriental*.

[16] Alice C. B., Silva G. A. A. B., Siqueira N. C. S., Mentz L. A. (1985). Levantamento fitoquímico de alguns vegetais utilizados na medicina popular do Rio Grande do Sul (Parte I). *Cadernos de Farmácia*.

[17] Arantes L. P. (2012). *Atividade antioxidante in vitro do extrato etanólico das folhas de Luehea divaricata Mart [M.S. thesis]*.

[18] Lopes E. (1990). *Avaliação das Atividades Biológicas de Luehea divaricata*.

[19] Bertucci A., Haretche F., Olivaro C., Vázquez A. (2008). Prospección química del bosque de galería del río Uruguay. *Revista Brasileira de Farmacognosia*.

[20] Vargas V. M., Guidobono R. R., Henriques J. A. (1991). Genotoxicity of plant extracts. *Memórias do Instituto Oswaldo Cruz*.

[21] Maraschin-Silva F., Aqüila M. E. A. (2006). Contribuição ao estudo do potencial alelopático de espécies nativas. *Revista Árvore*.

[22] Tanaka J. C. A., Vidotti G. J., Da Silva C. C. (2003). A new tormentic acid derivative from *Luehea divaricata* Mart. (Tiliaceae). *Journal of the Brazilian Chemical Society*.

[23] Choi H. J., Eun J. S., Kim B. G. (2006). Vitexin, an HIF-1*α* has anti-metastatic potential in PC12 cells. *Molecular Cell Biology*.

[24] Kim J. H., Lee B. C., Kim J. H. (2005). The isolation and antioxidative effects of vitexin from *Acer palmatum*. *Archives of Pharmacal Research*.

[25] Hsum Y. W., Yew W. T., Hong P. L. V. (2011). Cancer chemopreventive activity of maslinic acid: suppression of COX-2 expression and inhibition of NF-KB and AP-1 activation in raji cells. *Planta Medica*.

[26] Li C., Yang Z., Zhai C. (2010). Maslinic acid potentiates the anti-tumor activity of tumor necrosis factor *α* by inhibiting NF-*κ*B signaling pathway. *Molecular Cancer*.

[27] Bieski I. G. C., Santos F. R., de Oliveira R. M. (2012). Ethnopharmacology of medicinal plants of the pantanal region (Mato Grosso, Brazil). *Evidence-Based Complementary and Alternative Medicine*.

[28] Siqueira M. G. (2006). *Atividade antiulcerogênica do extrato bruto hidroalcoólico da Luehea divaricata Martus et Zuccarini [M.S. thesis]*.

[29] Walker C. I. B., Zanetti G. D., Ceron C. S. (2008). Morfoanatomia e Histoquimica das folhas de *Luehea divaricata* Mart. *Latin American Journal of Pharmacy*.

[30] da Silva D. A., Alves V. G., Franco D. M. M. (2012). Antiproliferative activity of *Luehea candicans* Mart. et Zucc. (Tiliaceae). *Natural Product Research*.

[31] Hegnauer R. (1973). *Chemotaxonomie der Pllanzen. Band 6*.

[32] Rosa E. A., Silva C. C., Oliveira C. M. A. Estudo fitoquímico preliminar da espécie vegetal Luehea grandiflora (Tiliaceae).

[33] Saénz J. A., Nassar M. C. (1968). Phytochemical screening of Costa Rica plants: alkaloid analysis III. *Revista de Biologia Tropical*.

[34] Coe F. G., Parikh D. M., Johnson C. A. (2010). Alkaloid presence and brine shrimp (Artemia salina) bioassay of medicinal species of eastern Nicaragua. *Pharmaceutical Biology*.

[35] Barbosa D. F. S., Reed E. Caracterização química das drogas obtidas a partir das folhas do açoita-cavalo.

[36] Sáenz J. A., Nassar M. (1970). Phytochemical screening of Costa Rican plants: alkaloid analysis. IV. *Revista de Biologia Tropical*.

[37] Calixto Júnior J. T., Morais S. M., Martins C. G. (2015). Phytochemical analysis and modulation of antibiotic activity by *Luehea paniculata* Mart. & Zucc. (Malvaceae) in multiresistant clinical isolates of *Candida* spp.. *BioMed Research International*.

[38] Alves V. G., Vandresen F., Rosa E. A. (2013). *Triterpenos e Atividade Antiproliferativa de Luehea paniculata (Tiliaceae)*.

[39] Silva D. A. (2004). *Estudo Químico e Avaliacão de Atividade Antifúngica e Antiproliferativa da Espécie Luehea candicans MART et ZUCC, (Tiliaceae) [M.S. thesis]*.

[40] Müller J. B. (2006). *Avaliação das atividades antimicrobiana, antioxidante e antinociceptiva das folhas da Luehea divaricata Martius [M.S. thesis]*.

[41] Bessa N., Borges J., Beserra F. (2013). Prospecção fitoquímica preliminar de plantas nativas do cerrado de uso popular medicinal pela comunidade rural do assentamento vale verde—Tocantins. *Revista Brasileira de Plantas Medicinais*.

[42] Longhi R. A. Lista das árvores: árvores e arvoretas do sul.

[43] Ritter M. R., Sobierajski G. R., Schenkel E. P., Mentz L. A. (2002). Plantas usadas como medicinais no município de Ipê, RS, Brasil. *Revista Brasileira de Farmacognosia*.

[44] Carvalho P. E. R. (2006). *Espécies Arbóreas Brasileiras*.

[45] Degen R., Soria N., Ortiz M. (2005). Problemática de nombres comunes de plantas medicinales comercializadas en Paraguay. *Dominguezia*.

[46] Carvalho J. E. (2006). Atividade antiulcerogênica e anticâncer de produtos naturais e de síntese. *Multiciência*.

[47] Monteles R., Pinheiro B. U. C. (2007). Plantas medicinais em um quilombo maranhense: uma perspectiva etnobotânica. *Revista de Biologia e Ciências da Terra*.

[48] Basualdo I., Soria N. (1996). Farmacopea Herbolaria Paraguaya: especies de la medicina Folklrica utilizada para combatir enfermedades del aparato respiratorio. *Rojasiana*.

[49] Maffei B. R. A. (1969). *Plantas Medicinales*.

[50] Chiriani C. H. B. (1982). *La Vuelta a los Vegetales—Tratado Moderno de Fitoterapia*.

[51] Freire F. W. (1933). Plantas medicinais brasileiras. *Boletin Agricola*.

[52] González M., Lombardo A., Vallarino A. J. Plantas de la medicina vulgar del Uruguay.

[53] Toursarkissian M. (1980). *Plantas Medicinales de la Argentina*.

[54] Alice C. B., Vargas V. M. F., Silva G. A. A. B. (1991). Screening of plants used in south Brazilian folk medicine. *Journal of Ethnopharmacology*.

[55] Roig Y., Mesa J. T. (1945). *Plantas Medicinales Aromáticas o Venenosas de Cuba*.

[56] Reitz R. (1950). Plantas medicinais de Santa Catarina. *Anais Botanicos do Herbário Barbosa Rodrigues*.

[57] da Rosa R. L., Nardi G. M., Januário A. G. F. (2014). Anti-inflammatory, analgesic, and immunostimulatory effects of *Luehea divaricata* Mart. & Zucc. (Malvaceae) bark. *Brazilian Journal of Pharmaceutical Sciences*.

[58] Alice C. B., Siqueira N. C. S., Mentz L. A. (1995). *Plantas Medicinais de Uso Popular: Atlas Farmacognóstico*.

[59] Saggesi D. (1959). *Yerbas Medicinales Argentinas*.

[60] Lahitte H. B., Hurrell M. J. B., Jankowski L. (1998). *Plantas Medicinales Rioplatenses*.

[61] Alonso J., Desmarchelier C. (2005). *Plantas Autóctonas Medicinales de la Argentina*.

[62] Zacchino S., Santecchia C., López S. (1998). *In vitro* antifungal evaluation and studies on mode of action of eight selected species from the Argentine flora. *Phytomedicine*.

[63] Montovani P. A. B., Gonçalves A. C., Moraes A. (2009). Atividade antimicrobiana do extrato de Açoita-cavalo (*Luehea sp*.). *Revista Brasileira de Agroecologia*.

[64] Coelho De Souza G., Haas A. P. S., Von Poser G. L., Schapoval E. E. S., Elisabetsky E. (2004). Ethnopharmacological studies of antimicrobial remedies in the south of Brazil. *Journal of Ethnopharmacology*.

[65] Marques M. C. S., Hamerski L., Garcez F. R. (2013). *In vitro* biological screening and evaluation of free radical scavenging activities of medicinal plants from the Brazilian Cerrado. *Journal of Medicinal Plant Research*.

[66] Bianchi N. R. (1996). Estudo da toxicidade de *Luehea divaricata*. *Revista Brasileira de Farmácia*.

[67] Rauber C., Mello F. B., Mello J. R. B. (2006). Avaliação toxicológica pré-clínica do fitoterápico contendo *Aristolochia cymbifera, Plantago major, Luehea grandiflora, Myrocarpus frondosus, Piptadenia colubrina* (Cassaú Composto) em ratos Wistar. *Acta Scientiarum Veterinária*.

[68] Souza S. A. M., Cattelaia L. V., Vargas D. P. (2005). Efeitos de extratos aquosos de plantas medicinais nativas do Rio Grande do Sul sobre a germinação de sementes de alface. *Biologia & Saúde*.

[69] Bighetti A. E., Ant M. A., Possent A., Antônio M. A. (2004). Efeitos da administração aguda e subcrônica da *Luehea divaricata* Martus et Zuccarini. *Lecta*.

[70] Felício L. P., Silva E. M., Ribeiro V. (2011). Mutagenic potential and modulatory effects of the medicinal plant *Luehea divaricata* (Malvaceae) in somatic cells of Drosophila melanogaster: SMART/wing. *Genetics & Molecular Research*.

